# Acknowledgement to Reviewers of *Plants* in 2014

**DOI:** 10.3390/plants4010027

**Published:** 2015-01-08

**Authors:** Plants Editorial Office

**Affiliations:** MDPI AG, Klybeckstrasse 64, CH-4057 Basel, Switzerland

The editors of *Plants* would like to express their sincere gratitude to the following reviewers for assessing manuscripts in 2014:

Abe, Jun
Anderson, Charles
Angelini, Riccardo
Bakkeren, Guus
Belin, Christophe
Binder, Brad
Bonhomme, Vincent
Bourgaud, Frederic
Brinch-Pedersen, Henrik
Brusslan, Judy
Carter, Clay
Chastain, Chris
Clark, Greg
Cosgrove, Daniel
Coyne, Clarice
D’Auria, Maurizio
Denecke, Jurgen
Diaz, Isabel
D’Ovidio, Renato
Dulormne, Maguy
Egea, Marcos
Finlayson, Scott
Franks, Robert
Frigerio, Lorenzo
Giraud, Tatiana
Goldman, Jason
Hanaoka, Mitsumasa
Harris, Philip
Hasegawa, Mike
Hause, Bettina
Hendrickson, John
Holuigue, Loreto
Horvath, David
Huang, Wen-Lii
Hussey, Patrick
Iakimova, Elena
Ishizaki, Takuma
Israel, Dan
Iwai, Hiroaki
Jiang, Liwen
Kasahara, Hiroyuki
Kinugasa, Toshihiko
Kiss, John
Knox, Paul
Krzymowska, Magdalena
Le Hir, Rozenn
Li, Jinyun
Li, Shundai
van der Linden, Gerard
Link, Gerhard
Lütz-Meindl, Ursula
Luu, Doan-trung
Ma, Wei
Mano, Yoshihiro
Mattoo, Autar
Maurel, Christophe
McLean, J. Paul
Medina, Joaquín
Mercado, José
Montgomery, Beronda
Odjakova, Mariela
Ó’Maoiléidigh, Diarmuid
Papini, Alessio
Parish, Roger
Pattathil, Sivakumar
Pauly, Markus
Pimpl, Peter
Quint, Marcel
Raboy, Victor
Recio, Antonio Serrato
Ribalta, Federico
Roberts, Jeremy
Rodríguez-Rosales, Maria Pilar
Romeis, Tina
di Sansebastiano, Gian-Pietro
Santino, Angelo
Sasaki-Sekimoto, Yuko
Satake, Akiko
Scherer, Günther
Shirsat, Anil Harishchandra
Solito, Egle
Sparkes, Imggen
Steinbach, Yvonne
Stepanova, Anna
Stotz, Henrik
Strader, Lucia
Taira, Suvi
Tazoe, Youshi
Tivendale, Nathan
Tosi, Paola
Ulvskov, Peter
van Cutsem, Pierre
van Doorn, Wouter
Veatch-Blohm, Maren
Veneault-Fourrey, Claire
Venema, Kees
Vinatzer, Boris
Wasteneys, Geoffrey
Wasternack, Claus
Yano, Sachiko
Žárský, Viktor

